# A hybrid framework for notebook market analysis: Integrating social media sentiment mining with expert knowledge for feature prioritization

**DOI:** 10.1371/journal.pone.0342067

**Published:** 2026-02-09

**Authors:** Mehrdad Maghsoudi, Mohammadreza Bakhtiari, Hamidreza Bakhtiari

**Affiliations:** 1 Management and Accounting Faculty, Shahid Beheshti University, Tehran, Iran; 2 Department of Electrical and Computer Engineering, University of Tehran, Tehran, Iran; 3 K.N. Toosi University of Technology, Tehran, Iran; CCET: Chandigarh College of Engineering and Technology, INDIA

## Abstract

The increasing complexity of consumer preferences in the notebook market requires advanced methodologies to effectively analyze user sentiments and prioritize product features for strategic decision-making. Traditional market research methods often fail to capture real-time, spontaneous consumer feedback while lacking integration with expert knowledge of actual purchasing behavior. This study introduces a novel hybrid framework that systematically combines aspect-based sentiment analysis (ABSA) of social media data with expert evaluations to bridge the gap between expressed consumer preferences and actual purchase drivers. The methodology analyzes 329,091 Twitter posts from January 2023 to June 2024, covering seven major notebook brands. Using the PyABSA framework, consumer sentiments toward 16 key notebook attributes are extracted and analyzed. Expert evaluations, conducted through fuzzy logic defuzzification, assign importance weights based on observed purchasing patterns, with features subsequently ranked using the TOPSIS multi-criteria decision-making method. Findings reveal that price, display quality, CPU performance, RAM capacity, and design constitute the most influential factors in notebook purchasing decisions. Negative sentiments concentrate on cooling systems, battery chargers, and warranty services, indicating critical improvement areas. Brand-specific analysis demonstrates that display quality ranks highest for Lenovo, while price dominates concerns for Dell, HP, and Microsoft, validating distinct market positioning strategies. By integrating machine learning-based sentiment analysis with structured expert knowledge, this research provides manufacturers with a quantifiable, actionable framework for optimizing product development and marketing strategies. The methodology enables companies to prioritize genuinely purchase-influencing features while addressing critical pain points, enhancing competitive positioning in dynamic markets. Future research should expand to cross-cultural consumer behavior analysis and real-time sentiment tracking systems.

## 1. Introduction

The notebook computer industry has become a cornerstone of the global technology market, driven by relentless innovation, expanding consumer needs, and intense competition [1,2]. As diverse user groups—including students, professionals, gamers, and creative practitioners—seek devices tailored to their specific requirements, manufacturers are compelled to identify, understand, and prioritize a multitude of product features. These features range from processing power and graphics performance to portability, design aesthetics, battery life, and overall user experience [3].

Recent progress in sentiment analysis has presented novel opportunities to refine our understanding of user preferences, with Aspect-Based Sentiment Analysis (ABSA) emerging as a particularly promising approach [4]. Although ABSA has been employed to examine sentiments in domains such as mobile phones [5] and restaurant reviews [6], its use in the notebook sector remains less explored. Notably, existing research often focuses on a single source of user feedback, such as historical product reviews, and overlooks real-time inputs from social media or the added value of expert judgment. This gap leaves a notable blind spot: manufacturers are unable to systematically integrate both consumer voices and expert evaluations when making decisions about product development and marketing strategies [7].

Furthermore, current market analysis strategies in the notebook industry suffer from three significant shortcomings. First, conventional techniques—such as surveys, focus groups, and basic sentiment assessments—may fail to capture the spontaneous, unstructured, and rapidly changing nature of consumer discourse on social media [8,9]. Second, sentiment analysis approaches commonly operate in isolation from the contextual expertise of industry professionals, resulting in assessments that may overlook the nuanced importance of specific features [10–13]. Third, a lack of comprehensive methodologies that fuse quantitative sentiment analysis with qualitative expert insights has prevented the notebook sector from establishing systematic models for accurate feature prioritization [14].

To address these challenges, a novel hybrid framework merges three essential components: (1) aspect-based sentiment analysis of social media data using the PyABSA framework, (2) expert-based evaluations facilitated by fuzzy logic, and (3) feature prioritization through the TOPSIS method. By integrating these components, the framework provides a robust mechanism for translating unstructured consumer feedback into clear, actionable insights. This data-driven methodology offers a more dynamic understanding of user sentiments and a structured way to incorporate these insights into product development and marketing decisions [15].

Our research makes several distinct contributions to the literature. First, by integrating automated sentiment analysis with expert evaluation, we deliver a more granular perspective on consumer preferences, allowing manufacturers to distinguish between essential and peripheral product features more effectively. Second, we introduce a rigorous process for mapping consumer sentiments—both positive and negative—to purchase intention through the combined application of fuzzy logic and TOPSIS. Third, we present empirical findings that detail how different notebook brands are perceived across multiple product attributes, thereby furnishing practical guidelines for marketing campaigns and research and development efforts.

In line with these insights, our study targets three principal objectives: (1) to identify and analyze user sentiments regarding key notebook components across different brands, (2) to evaluate how these sentiments, when combined with expert knowledge, influence purchase decisions, and (3) to develop a prioritized framework that synthesizes consumer feedback and expert perspectives for guiding both marketing and research and development initiatives. By comprehensively examining consumer discourse and industry expertise, we seek to provide a blueprint for manufacturers aiming to stay ahead in a market shaped by rapidly shifting demands and a broad spectrum of user preferences.

The subsequent sections are structured to facilitate an in-depth understanding of our contribution and its broader implications. Section 2 reviews the existing literature relevant to sentiment analysis, consumer electronics, and multi-criteria decision-making methods, setting the theoretical stage for the hybrid approach. Section 3 describes the methodology and data collection procedures in detail. Section 4 presents the empirical findings, while Section 5 interprets these results and situates them within the context of notebook market strategies. Section 6 concludes with the key insights and contributions, and Section 7 discusses limitations and potential avenues for future investigation.

## 2. Literature review

### 2.1. Consumer preferences in the notebook market

Consumer preferences in the notebook market have evolved significantly over the past decade, driven by changing usage patterns and technological advances. Previous research has identified price, performance, and portability as traditional decision factors. However, recent studies suggest that consumers increasingly value specific features differently based on their use cases [16].

Student consumers prioritize battery life and weight, reflecting their need for all-day usage and campus mobility. Professional users focus on display quality and keyboard comfort, essential for extended work sessions and productivity tasks. Gaming enthusiasts emphasize GPU performance and cooling systems, critical for sustained high-performance computing [17].

Market segmentation research reveals distinct preference patterns across demographics. Younger consumers (18–25) show higher sensitivity to design aesthetics and brand image, while older professionals (35–50) prioritize reliability and enterprise features. Geographic variations also influence preferences, with Asian markets showing stronger preference for compact designs compared to North American consumers who favor larger screens. In studies comparing students vs working professionals, [18] show similar base brand and screen-size preferences, though trade-offs among features differ.

Price sensitivity varies significantly across segments. While budget-conscious students demonstrate high price elasticity, business users show willingness to pay premiums for durability and support services [19]. This heterogeneity in price perception challenges manufacturers to develop differentiated pricing strategies across product lines.

Despite these insights, most existing research relies on surveys or sales data, missing the real-time, unsolicited opinions consumers express on social media. Traditional market research methods often suffer from response bias and lack the spontaneity of natural consumer discourse. The recent work [20] applies aspect-based sentiment analysis to laptop commentary and shows how feature-level sentiment differs across markets. Moreover, ABSA has been successfully applied in related smart device domains [21] and in e-commerce review classification [22]. In the methodological literature, advanced techniques such as graph convolutional joint models [23] and attention-over-attention networks [24] further motivate our methodological choice.

This gap motivates our use of aspect-based sentiment analysis to capture authentic consumer preferences from online discussions.

### 2.2. Overview of aspect-based sentiment analysis

Sentiment Analysis (SA), also known as Opinion Mining, is the computational study of people’s opinions, attitudes, and emotions towards entities such as products, events, or topics [4,20]. This field has gained substantial attention due to its ability to extract valuable insights from user-generated content. SA encompasses various techniques and approaches, including machine learning, lexicon-based, and hybrid methods, to classify the polarity of opinions at different levels: document, sentence, and aspect [25,26]. Document-level SA focuses on determining the overall sentiment of a text, while sentence-level SA aims to classify the sentiment of individual sentences [27]. However, these approaches often lack the granularity required for detailed analysis, leading to the development of aspect-level SA, which identifies sentiments towards specific aspects within a text. The applications of SA are vast, ranging from product reviews and stock market analysis to political debates and social media monitoring, making it a crucial tool for businesses and researchers alike to understand and predict trends and consumer behavior [28].

Aspect-based sentiment Analysis (ABSA) is a sophisticated sub-field of sentiment analysis that focuses on identifying and categorizing opinions about specific aspects within text data [29,30]. This methodology has gained significant attention due to its ability to provide detailed insights into user-generated content, making it valuable across various domains. This literature review is divided into two sections: the first discusses the models and methods used in ABSA, while the second explores its real-world applications.

### 2.3. Technical advances in ABSA methods

Aspect-Based Sentiment Analysis (ABSA) has evolved significantly, with various methodologies proposed to enhance its accuracy and efficiency. Traditional supervised learning approaches, such as those by Pannala et al. (2016), leverage machine learning algorithms like Support Vector Machines (SVM) and Maximum Entropy (ME) classifiers to classify sentiments in product reviews [31]. Their research underscores the importance of preprocessing steps, such as normalizing text and handling informal language, to improve sentiment analysis accuracy [32].

Transitioning from traditional methods, deep learning techniques have brought substantial advancements to ABSA [33]. Do et al. (2019) provide a comprehensive review of deep learning models, highlighting the superiority of Convolutional Neural Networks (CNNs) and Recurrent Neural Networks (RNNs), including Long Short-Term Memory (LSTM) networks, in capturing both syntactic and semantic features of text [34]. Xue and Li (2018) introduced Gated Convolutional Networks (GCNs) with Aspect Embedding (GCAE), demonstrating higher accuracy and reduced training time compared to LSTM-based models [35].

Pre-trained language models like BERT have also been adapted for ABSA, as shown by Xu et al. (2019). They proposed a methodology to adapt BERT’s pre-trained weights for domain-specific nuances in product reviews, achieving significant improvements in aspect extraction and sentiment classification tasks [36]. Phan and Ogunbona (2020) further enhanced these models by integrating syntactical information into contextualized embeddings, improving both aspect extraction (AE) and aspect sentiment classification (ASC) [37].

Unsupervised and semi-supervised methods have also been explored to address data scarcity in ABSA. Ozyurt and Akcayol (2020) proposed Sentence Segment Latent Dirichlet Allocation (SS-LDA) for short texts, effectively improving the precision, recall, and F-score of aspect extraction tasks without the need for annotated training data [38]. García-Pablos, Cuadros, and Rigau (2017) introduced W2VLDA, a system combining guided topic modeling with continuous word embeddings, showing competitive performance across multiple domains and languages with minimal supervision [39].

Hierarchical models have proven effective in leveraging contextual dependencies. Ruder et al. (2016) proposed a hierarchical bidirectional LSTM model to capture interdependencies of sentences within a review, improving sentiment classification accuracy compared to non-hierarchical baselines [40]. Liu et al. (2018) introduced Cabasc, a content attention-based model that incorporates sentence-level and context attention mechanisms, achieving higher accuracy in sentiment classification tasks [41].

**Fig 1:**
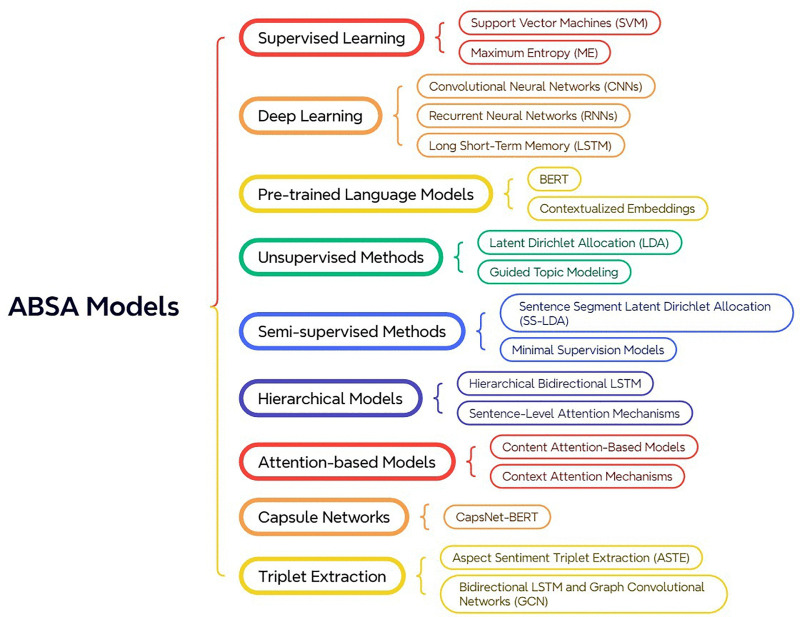
ABSA Models and Methods.

Innovative frameworks have been developed to enhance ABSA. Jiang et al. (2019) introduced the Multi-Aspect Multi-Sentiment (MAMS) dataset and models like CapsNet-BERT, which leverage capsule networks to model complex relationships between aspects and contexts [42]. Peng et al. (2020) proposed Aspect Sentiment Triplet Extraction (ASTE) to extract comprehensive sentiment information, including aspect, sentiment polarity, and opinion reason, using Bidirectional LSTM and Graph Convolutional Networks (GCN) [43]. Several surveys provide a detailed overview of ABSA methodologies. Nazir et al. (2020) and Zhang et al. (2023) offer comprehensive reviews of tasks, methods, and challenges in ABSA, highlighting the advancements brought by pre-trained language models and proposing future research directions [44,45]. Fig 1 shows a summary of these methods.

### 2.4. Applications and domain-specific implementations

Aspect-based sentiment Analysis (ABSA) has found extensive applications across various domains, offering nuanced insights into user-generated content and enhancing decision-making processes. In the hospitality industry, Sann and Lai [46] leveraged ABSA to explore service failures in hotel reviews, uncovering significant cultural differences in guest complaints. This study highlighted the utility of ABSA in identifying areas for service improvement and tailoring responses to meet diverse guest expectations, thereby enhancing overall customer satisfaction. Similarly, Tran, Ba [47] employed ABSA to analyze hotel reviews in Ho Chi Minh City, Vietnam, combining aspect term extraction and polarity classification with topic modeling. Their findings provided actionable insights for hotel managers to improve service quality by targeting specific areas identified through sentiment analysis.

In the public sector, Alqaryouti, Siyam [48] demonstrated the potential of ABSA in enhancing smart government applications. By integrating domain-specific lexicons and rule-based techniques with Support Vector Machine (SVM) models, they effectively extracted significant aspects from user feedback, offering valuable insights for improving public services. The healthcare domain has also benefited from ABSA, as evidenced by Gräßer, Kallumadi [49], who analyzed drug reviews to enhance pharmacovigilance and clinical decision support systems. Their approach facilitated the monitoring of drug safety and efficacy through user-generated content, highlighting the importance of patient feedback in the pharmaceutical industry.

In the realm of mobile applications, Alturaief, Alturaief, Aljamaan [50] introduced the AWARE dataset, designed for requirements elicitation from app reviews. Their work demonstrated the practical applications of ABSA in the mobile app industry by providing detailed insights into user experience, which in turn supports continuous app quality enhancement and user-centered development. Similarly, in the e-commerce sector, Rodrigues and Chiplunkar [51] explored ABSA on product reviews, focusing on electronic gadgets. Their system effectively identified relevant aspects and classified sentiments, providing granular insights into customer opinions that drive product development and enhance customer satisfaction.

The education sector has also seen applications of ABSA, as Sivakumar and Reddy [52] utilized this technique to analyze student feedback, aiming to improve educational services. By categorizing opinions based on various aspects such as teaching and facilities, their study provided actionable insights for educational institutions to enhance the overall student experience. In the context of Arabic language resources, Al-Ayyoub, Gigieh [53] developed the Arabic Laptop Reviews (ALR) dataset, highlighting the challenges and potential of ABSA in analyzing Arabic user-generated content. Their work contributed significantly to the advancement of Arabic Natural Language Processing (NLP) and sentiment analysis.

Gupta and Ekbal [6] participated in the SemEval-2014 shared task, focusing on ABSA for restaurant and laptop reviews. Their approach employed various machine learning algorithms to extract aspect terms and classify sentiments, demonstrating the effectiveness of ABSA in mining relevant information from online reviews and providing insights into customer opinions on specific product features. Yiran and Srivastava [5] further applied ABSA to mobile phone reviews, particularly focusing on the iPhone X. Their framework, which combined ABSA with Latent Dirichlet Allocation (LDA) for topic modeling, effectively extracted and weighed various product aspects, offering valuable implications for both consumers and manufacturers in the e-commerce domain.

Finally, Li, Bruce [54] applied ABSA to predict restaurant survival using customer-generated content from Yelp reviews. Their study demonstrated that aspect-based sentiment significantly improves the accuracy of survival predictions compared to models using only overall sentiment. This research provided valuable insights for restaurant owners and managers, suggesting that focusing on specific aspects of customer experience can enhance survival prospects and inform better strategic decisions in the highly competitive restaurant industry. Table 1 shows the Summary of ABSA applications in previous studies.

**Table 1:** Summary of ABSA applications in previous studies.

Domain	Year	Application	Key Findings
Restaurants	2014	SemEval-2014 ABSA for restaurant and laptop reviews	Employed machine learning algorithms to extract aspect terms and classify sentiments, demonstrating effectiveness in mining relevant information from reviews.
Education	2017	Student feedback analysis	Categorized opinions based on aspects like teaching and facilities, offering actionable insights to improve educational services.
Arabic NLP	2017	Arabic Laptop Reviews (ALR) dataset	Advanced Arabic Natural Language Processing (NLP) and sentiment analysis by addressing challenges in analyzing Arabic user-generated content.
E-commerce	2017	Product reviews analysis for electronic gadgets	Identified relevant aspects and classified sentiments, offering granular insights into customer opinions to drive product development and satisfaction.
Healthcare	2018	Analyzing drug reviews	Enhanced pharmacovigilance and clinical decision support systems by monitoring drug safety and efficacy through user feedback.
Public Sector	2019	Enhancing smart government applications	Integrated domain-specific lexicons and SVM models to extract significant aspects from user feedback, improving public services.
Mobile Phones	2019	iPhone X reviews	Combined ABSA with LDA for topic modeling, extracting, and weighing various product aspects, providing valuable implications for consumers and manufacturers.
Hospitality	2020	Analyzing service failures in hotel reviews	Identified cultural differences in complaints, highlighting areas for service improvement and tailored responses to enhance customer satisfaction.
Hospitality	2020	Hotel reviews analysis in Ho Chi Minh City	Combined aspect term extraction, polarity classification, and topic modeling to provide actionable insights for improving service quality.
Mobile Applications	2021	AWARE dataset for app reviews	Provided insights into user experience, supporting continuous app quality enhancement and user-centered development.
Restaurants	2023	Predicting restaurant survival using Yelp reviews	Demonstrated that aspect-based sentiment improves the accuracy of survival predictions, offering insights for enhancing customer experience and strategic decision-making.

### 2.5. Multi-criteria decision making: Fuzzy logic and TOPSIS

While ABSA provides granular insights into consumer sentiments, translating these sentiments into actionable business priorities requires robust decision-making frameworks capable of handling multiple criteria simultaneously. Multi-Criteria Decision Making (MCDM) methods have emerged as essential tools for synthesizing diverse inputs into prioritized recommendations [55].

Fuzzy logic, introduced by [56], offers a mathematical framework for handling uncertainty and imprecision inherent in human judgments. Unlike classical binary logic, fuzzy logic allows variables to have degrees of membership across multiple categories, making it particularly suitable for capturing expert opinions expressed in linguistic terms such as “high,” “medium,” or “low” [57]. In the context of market analysis, fuzzy logic enables the systematic conversion of qualitative expert assessments into quantitative values through defuzzification techniques such as the Center of Gravity method [58]. This capability is especially valuable when integrating subjective expert knowledge with objective data-driven insights, as demonstrated in applications ranging from risk assessment to product evaluation [59].

The Technique for Order of Preference by Similarity to Ideal Solution (TOPSIS), developed by Hwang and Yoon [60], has become one of the most widely adopted MCDM methods due to its computational simplicity and intuitive logic. TOPSIS ranks alternatives based on their geometric distance from positive and negative ideal solutions, effectively balancing multiple criteria to identify optimal choices [61]. The method has been successfully applied across diverse domains, including supplier selection, project prioritization, and consumer product evaluation [62]. Its ability to incorporate both benefit and cost criteria makes it particularly suitable for analyzing consumer preferences, where both positive sentiments (motivating purchase) and negative sentiments (deterring purchase) must be considered simultaneously.

Recent research has demonstrated the effectiveness of integrating sentiment analysis with MCDM approaches. [63] combined ABSA with graph-based classification to analyze consumer segmentation, showing how sentiment-driven insights can inform product development strategies. Similarly, hybrid frameworks that merge natural language processing with structured decision-making methods have proven valuable for transforming unstructured social media data into strategic recommendations [64]. These integrations address a fundamental limitation of standalone sentiment analysis: while ABSA captures what consumers express, MCDM methods help determine which expressions should guide business decisions based on their relative importance and impact.

The complementary nature of these methodologies forms the theoretical foundation for our hybrid framework. ABSA extracts feature-specific sentiments from consumer discourse, fuzzy logic captures and quantifies expert knowledge about purchase behavior, and TOPSIS synthesizes these inputs to generate prioritized recommendations that balance consumer voice with market realities.

Building upon the established understanding of consumer preferences in the notebook market, the proven capabilities of ABSA methodology, and the robust decision-making frameworks provided by fuzzy logic and TOPSIS, this article presents a novel integration that addresses critical gaps in current research. Unlike previous studies that either focus solely on stated preferences through surveys, rely on sentiment analysis without market context, or apply MCDM methods without real-time consumer input, our approach synthesizes these complementary methodologies into a unified framework. By combining ABSA’s ability to capture authentic consumer voice, fuzzy logic’s capacity to quantify expert knowledge, and TOPSIS’s systematic prioritization capabilities, we provide a more nuanced and actionable framework for notebook manufacturers. This hybrid methodology not only identifies what consumers say they want but also determines which expressed preferences genuinely drive purchasing decisions, offering a comprehensive blueprint for product development and marketing strategies in the competitive notebook market.

## 3. Research methodology

**Fig 2:**
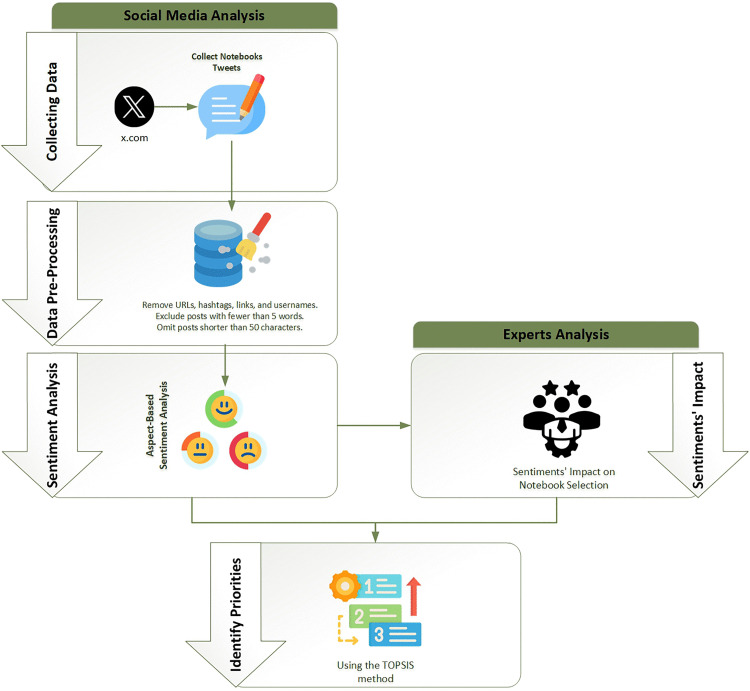
Research methodology.

The methodology for this research involves a multi-step process to analyze marketing priorities and research and development of notebook items using sentiment analysis of social networks. While ABSA captures what consumers express online, expert evaluations provide crucial context about which expressed sentiments translate into actual purchasing behavior, based on their direct observation of thousands of customer decisions. The entire process is depicted in Fig 2, which illustrates the integrated framework comprising two parallel analytical streams that converge to produce prioritized recommendations.

The left stream represents the Social Media Analysis pipeline, consisting of three sequential stages: (1) Collecting Data, gathering notebook-related tweets from X, detailed in Section 3.1; (2) Data Pre-Processing, cleaning and filtering the collected tweets by removing URLs, hashtags, links, and usernames, excluding posts with fewer than 5 words, and omitting posts shorter than 50 characters, as described in Section 3.2; and (3) Sentiment Analysis, applying Aspect-Based Sentiment Analysis using the PyABSA framework to extract feature-specific sentiments, elaborated in Section 3.3.

The right stream represents the Experts Analysis component, where industry professionals evaluate the impact of sentiments on notebook selection using fuzzy logic linguistic scales, as detailed in Section 3.4. This stream captures expert knowledge about which consumer sentiments translate into actual purchasing behavior.

The two streams converge at the Identify Priorities stage, where the TOPSIS multi-criteria decision-making method synthesizes consumer sentiments with expert-validated importance weights to produce a prioritized ranking of notebook features, as described in Section 3.4 (Identifying Priorities subsection). This integration ensures that the final recommendations reflect both authentic consumer voice and market-validated purchase drivers.

### 3.1. Data collection

In the initial phase of this research, we collect user-generated opinions about notebook products from the social media platform X (formerly Twitter). This platform is chosen due to its extensive user base and the richness of real-time content it provides [65,66]. Using the official X API and a structured, query-based approach, we retrieve English-language tweets posted over the past two years, focusing on seven major notebook brands analyzed in this study. A predefined set of feature-specific keywords is applied to ensure that only relevant tweets are extracted. let T denote the complete set of collected tweets, where each t ∈ T includes notebook-related feature terms along with corresponding timestamp information. These data represent spontaneous consumer feedback and sentiment toward various notebook attributes.

To ensure reproducibility and transparency, the entire dataset is made publicly available on Hugging Face (https://huggingface.co/datasets/MrbBakh/Twitter_Laptop), and all data processing and analytical scripts are shared on GitHub (https://github.com/Bakhtiarii/hybrid-absa-notebook-analysis). This open-access approach aligns with open science principles while complying with both the platform’s terms of service and ethical research standards.

### 3.2. Data pre-processing

The collected data undergoes a thorough pre-processing phase to ensure its quality and relevance for subsequent analysis. The steps involved in data pre-processing are:

- Removal of URLs, hashtags, links, and usernames: This step eliminates non-essential elements that do not contribute to sentiment analysis [67,68].
- Posts with fewer than 5 words are excluded to remove non-informative content, spam, and bot-generated posts that typically lack substantive information for sentiment analysis [68,69]. Short posts often contain only hashtags, mentions, or fragmented phrases that do not provide meaningful insights into user sentiment toward specific product features [70]. This 5-word threshold ensures that only posts with adequate contextual information are included for aspect-based sentiment analysis [20].
- Omission of posts shorter than 50 characters: This further ensures that only substantial and informative posts are considered for analysis [68].

These measures refine the dataset, making it a robust input for sentiment analysis.

### 3.3. Sentiment analysis

The sentiment analysis phase employs the PyABSA framework to analyze the pre-processed tweets. PyABSA is an open-source tool specifically designed for Aspect-Based Sentiment Analysis (ABSA), with extensive validation in research applications [71–73]. The implementation follows a three-stage process comprising aspect term extraction, sentiment classification, and model optimization.

In the aspect term extraction stage, the framework utilizes a fine-tuned BERT-based sequence labeling model to identify relevant features. Each tweet *t* is processed to extract aspect terms $A=\{a_{\text{1}},\;a_{\text{2}},\;\ldots ,\;a_{n}\}$, where $a_{i}$ represents specific notebook features mentioned. The extraction model leverages domain-specific vocabulary and contextual embeddings to improve accuracy in identifying relevant aspects within the text.

The sentiment classification stage processes each identified aspect individually, performing targeted sentiment analysis through contextual evaluation. For each identified aspect $a_{i}$, the framework classifies sentiment polarity as either positive (1) or negative (−1). This classification process utilizes sophisticated attention mechanisms to focus on aspect-relevant contexts within tweets.

PyABSA’s modular architecture supports up to 40 built-in models, enabling comprehensive sentiment analysis through sophisticated model ensemble techniques [71]. The framework demonstrates state-of-the-art performance in both aspect extraction and sentiment classification tasks. According to Yang and Li (2022), PyABSA’s efficient architecture provides quick training and instant inference while maintaining high accuracy, outperforming traditional models in multilingual and multi-domain scenarios [74,75].

To validate PyABSA’s performance on our specific notebook review dataset, we conducted evaluation on a held-out sample of 1,000 tweets randomly selected from our corpus. This subset was manually annotated by two independent researchers with expertise in sentiment analysis, achieving an inter-annotator agreement of 0.87 (Cohen’s kappa). The manual annotation process involved identifying aspect terms and their corresponding sentiment polarities (positive, negative) for each tweet. PyABSA’s performance on this validation set demonstrated strong results with a precision of 0.85, recall of 0.82, and F1-score of 0.83 for aspect-based sentiment classification. These metrics confirm the framework’s effectiveness in accurately extracting and classifying sentiments toward specific notebook features within our Twitter dataset, ensuring the reliability of our subsequent analysis.

### 3.4. Expert analysis

To understand the impact of positive and negative sentiments on notebook selection, we incorporate the opinions of industry experts. These experts serve as informed intermediaries who observe actual consumer purchasing behavior daily [76,77]. Unlike survey respondents who might state preferences theoretically, sales experts witness which features actually drive or prevent purchases. Their evaluations are based on thousands of real customer interactions, complaints, returns, and successful sales. Research has shown that sales professionals develop sophisticated mental models of consumer preferences through repeated exposure to purchase decisions, making them valuable proxies for understanding market behavior [78,79]. For instance, they observe customers willing to compromise on features they complain about (like fans) but unwilling to purchase when price or display don’t meet expectations. This experiential knowledge bridges the gap between stated preferences (captured by ABSA) and revealed preferences in actual purchasing decisions [80,81]. Experts evaluate the significance of each notebook feature by rating them based on the strengths and weaknesses observed from user sentiments. This evaluation is done using fuzzy logic, where features are rated on a five-point scale: “very low, low, medium, high, and very high.”

Fuzzy logic provides a robust mechanism to capture expert opinions, especially when dealing with subjective assessments [57]. In fuzzy logic, a variable can have a degree of membership in multiple sets simultaneously, described by membership functions [82]. These functions map the input space to a membership value between 0 and 1 [59].

In fuzzy logic, the aggregation of expert opinions results in fuzzy sets for each notebook feature. Defuzzification is the process of converting these fuzzy sets into crisp values [83]. The Center of Gravity (CoG) method, also known as the centroid method, is a commonly used defuzzification technique [58]. It calculates the center of the area under the curve of the membership function [84]. The formula for the CoG method is:


$$\text{COG}\;=\;\frac{\sum (\mu (x)*x)}{\sum (\mu (x))}$$

where: μ(x) is the membership function of the fuzzy set at value x and x is a value within the domain of the fuzzy set [85].

This method provides a single crisp value that represents the center of the area under the membership function curve, giving a balanced representation of the fuzzy set.

Identifying Priorities

In the final step, we prioritize the features of notebooks based on their impact on consumer purchase decisions using the TOPSIS method (Technique for Order of Preference by Similarity to Ideal Solution). TOPSIS is a powerful multi-criteria decision-making method that considers both positive and negative factors for prioritization [61,62].

The steps involved in the TOPSIS method are:

Step 1: make a fuzzy decision matrix with dimensions m*n to capture the perspectives of the individuals


$$D=[\begin{array}{c} \begin{array}{ccc} x_{\text{11}} & x_{\text{12}} & x_{\text{1}n}\\ . & . & .\\ x_{m\text{1}} & x_{m\text{2}} & x_{mn} \end{array} \end{array}]$$

Step 2: Normalization of the decision matrix:


$$r_{ij}=\frac{X_{ij}}{\sqrt{\sum_{i=\text{1}}^{m}X_{ij}^{\text{2}}}}$$

Step 3: make a weighted normalized Fuzzy Matrix V.

The creation of weighted matrices involves multiplying the normalized matrix by the weight of $v_{ij}=r_{ir}\times w_{\text{j}.}$. The matrix is as follows:


$$V=[\begin{array}{c} \begin{array}{ccc} v_{\text{11}} & v_{\text{12}} & v_{\text{1}n}\\ . & . & .\\ v_{m\text{1}} & v_{m\text{2}} & v_{mn} \end{array} \end{array}]$$

Step 4: Calculate Positive Ideal solution $A^{*}$ and Negative Ideal solution $A^{-}$.


$$A^{*}=\;((\text{Max}\;\;v_{ij}|\;\text{i}\;=\;\text{1},\text{2},\;\ldots ,\;\text{m},\;\forall_{j}\;\in \;J_{+}),\;(\text{Min}\;\;v_{ij}|\;\text{i}\;=\;\text{1},\text{2},\;\ldots ,\;\text{m},\;\forall_{j}\;\in \;J_{-}))$$

$$A^{-}=\;((\text{Max}\;\;v_{ij}|\;\text{i}\;=\;\text{1},\text{2},\;\ldots ,\;\text{m},\;\forall_{j}\;\in \;J_{+}),\;(\text{Min}\;\;v_{ij}|\;\text{i}\;=\;\text{1},\text{2},\;\ldots ,\;\text{m},\;\forall_{j}\;\in \;J_{-}))$$

Step 5: Calculate the sum of components distance from the positive ideal and negative ideal values.


$$d_{i}^{+}=\sum_{j=\text{1}}^{n}d(v_{ij},\;v_{j}^{*}),\;\;\;i=\text{1},\;\text{2},\;...,\;m$$

$$d_{i}^{-}=\sum_{j=\text{1}}^{n}d(v_{ij},\;v_{j}^{-}),\;\;\;i=\text{1},\;\text{2},\;...,\;m$$

Step 6: Calculate the similarity to the ideal option. To achieve this, the Closeness coefficient (CCi) should be calculated. The option with the highest CCi is better.


$$C_{i}^{*}=\frac{{\;d}_{i}^{-}}{{\;d}_{i}^{-}+{\;d}_{i}^{+}}$$

This method helps in identifying which features are most influential in driving purchase decisions and which negative aspects deter consumers from buying, thereby providing a clear set of priorities for marketing and R&D efforts.

### 3.5. Participant consent

This study did not involve direct human participation, clinical trials, or the collection of personal or sensitive information from individuals. All data analyzed were derived from publicly available, anonymized Twitter posts and non-identifiable expert responses collected through a voluntary online questionnaire. Expert participants contributed general, non-personal evaluations related to product features and were not asked to provide any personal or sensitive information. As such, no written or verbal informed consent was required or obtained, and no minors were involved in the study. The study complies with ethical guidelines for research using anonymized and non-identifiable data, and the nature of the data collection did not necessitate ethical review or consent procedures.

## 4. Results

### 4.1. Data collection

The primary dataset for this study comprises laptop reviews collected from Twitter, covering seven well-known laptop brands: Asus, Acer, Apple, Dell, HP, Lenovo, and Microsoft. The aim is to gather a comprehensive set of user opinions and sentiments regarding various laptop models and features. To compile the dataset, a query-based search methodology was utilized. This involved creating a list of keywords relevant to laptops in general and specific to each brand. By leveraging these query lists, tweets containing at least one of the specified keywords were identified. For instance, keywords such as “laptop,” “Asus,” “MacBook,” and “CPU” were used to filter relevant reviews. For each brand, specific product names and model identifiers were included to ensure a broad and representative sample of reviews. The query lists for each brand are as Table 2.

**Table 2:** Query list.

Brand	Query List
Asus	“Asus AND laptop,” “zenbook,” “vivobook,” “Flow AND Asus,” “Zephyrus AND Asus,” “TUF AND laptop,” “Chromebook AND Asus,” “ProArt StudioBook,” “asus x,” “ExpertBook,” “ROG AND laptop”
Acer	“Acer AND laptop,” “Aspire AND laptop,” “ConceptD,” “Extensa AND acer,” “Swift AND acer,” “Spin AND acer,” “Nitro AND acer,” “Predator AND laptop,” “TravelMate AND acer,” “Chromebook AND acer,” “Ferrari AND acer”
Apple	“Apple AND laptop,” “MacBook”
Dell	“Dell AND laptop,” “Studio AND Dell,” “Inspiron AND Dell,” “XPS AND Dell,” “Vostro AND Dell,” “Latitude AND Dell,” “Precision AND Dell,” “Alienware AND Dell,” “Chromebook AND Dell,” “Dell G”
HP	“HP AND laptop,” “Victus AND laptop,” “Stream AND HP,” “Spectre AND HP,” “Envy AND HP,” “Pavilion AND HP,” “Omen AND laptop,” “EliteBook,” “ProBook,” “ZBook,” “Chromebook AND HP,” “Dragonfly AND HP,” “Elite Dragonfly,” “Dev One”
Lenovo	“Lenovo AND laptop,” “ThinkPad,” “IdeaPad,” “Legion AND Lenovo,” “Yoga AND Lenovo,” “ThinkBook,” “Chromebook AND Lenovo”
Microsoft	“Surface Pro 10,” “Surface Pro 11,” “Surface Laptop 6,” “Surface Laptop Go 3,” “Surface Laptop Studio 2,” “Surface Laptop 7,” “Surface Studio,” “Surface Duo,” “Surface Go,” “Surface Book,” “Surface & neo,” “Surface & pro,” “Surface AND laptop,” “Microsoft AND surface”

**Fig 3:**
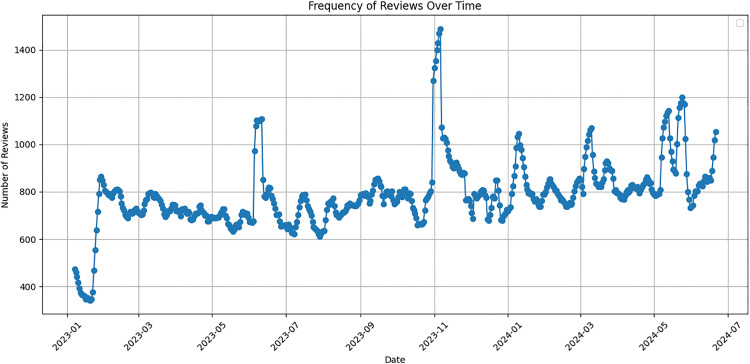
Tweet collection frequency from 329,091 notebook reviews spanning January 2023 to June 2024.

The tweets were collected over a period spanning from January 1, 2023, to June 22, 2024. This range was chosen to ensure the data reflects recent trends and opinions about the laptops from the specified brands. Initially, a total of 422,649 tweets were collected. To ensure the quality and reliability of the dataset, only tweets that had at least one “like” were included. This criterion helped in filtering out potentially less relevant or less impactful reviews. To visualize the frequency of reviews over the specified period, a time trend chart was created. Fig 3 illustrates the frequency of reviews from January 2023 to June 2024, revealing several distinct temporal patterns. The low volume observed in early January 2023 (approximately 350–400 daily tweets) reflects the typical post-holiday period when consumer discussions about technology purchases temporarily decline following the peak shopping season. Activity gradually stabilized to approximately 700–800 daily tweets throughout the first half of 2023. A notable peak occurred around November 2023, reaching approximately 1,500 daily tweets, nearly double the baseline activity. This surge coincides with the major shopping events of Black Friday and Cyber Monday, when consumer interest in notebook purchases intensifies significantly due to promotional pricing and holiday gift-buying. Secondary peaks visible around June 2023 and May-June 2024 align with back-to-school shopping periods and mid-year product refresh cycles. These temporal patterns validate the representativeness of our dataset, as it captures both routine consumer discourse and peak purchasing consideration periods.

### 4.2. Data pre-processing

Data pre-processing is a crucial step in ensuring the quality and reliability of the dataset. For this study, the primary focus was on cleaning and refining the data collected from Twitter to remove noise and enhance its usability for Aspect-Based Sentiment Analysis (ABSA). The initial dataset consisted of 422,649 tweets, which were subsequently filtered and cleaned to produce a final dataset of 329,091 reviews. The pre-processing of the dataset involved several key steps:

1. Conversion to String Format: All entries in the reviews were converted to strings to maintain consistency and facilitate further processing.
2. Handling Missing Values: Any missing values (NaNs) in the reviews were replaced with empty strings to prevent issues during text analysis.
3. Removal of Duplicates: Duplicate reviews, where the same review appeared more than once, were identified and removed to ensure that each review was unique. This step helped in reducing redundancy in the dataset.
4. Text Cleaning: The text of the reviews underwent several cleaning processes:
  a) Lowercasing: All text was converted to lowercase to maintain uniformity.
  b) Removal of URLs: Links within the tweets were removed to eliminate irrelevant content.
  C) Removal of Mentions and Hashtags: Mentions (e.g., @username) and hashtags (e.g., #laptop) were removed to focus on the substantive content of the reviews.
5. Filtering Short Reviews: To enhance the quality of the dataset, reviews with fewer than 5 words or fewer than 50 characters were filtered out. This criterion ensured that only reviews with sufficient content were included for analysis.

The elimination of reviews was guided by specific criteria to improve the dataset’s relevance and quality. Short reviews that were too brief were deemed less informative and were excluded, while redundant reviews were removed to avoid skewing the analysis. After the data pre-processing steps, the dataset was refined to 329,091 reviews. This cleaned dataset provided a robust foundation for performing ABSA, ensuring that the analysis was based on relevant and high-quality data.

Following the data pre-processing steps, we conducted a dataset quality assessment to ensure balanced representation across key dimensions. The final dataset demonstrates reasonable distribution across the seven notebook brands with no significant brand bias that could skew sentiment analysis results. Temporal distribution analysis reveals consistent data collection across the study period, ensuring that our findings are not influenced by seasonal variations or specific events. The aspect-based sentiment distribution shows balanced representation across the identified features, with core performance features accounting for the majority of sentiment expressions, which aligns with expected consumer focus areas. Our current assessment demonstrates sufficient data quality and representativeness for reliable aspect-based sentiment analysis across notebook brands and features.

### 4.3. Aspect analysis implementation

Aspect-Based Sentiment Analysis (ABSA) is a fine-grained technique used to determine the sentiment expressed towards specific aspects or features within a text. Unlike general sentiment analysis, which provides an overall sentiment for a piece of text, ABSA identifies sentiments related to particular entities or attributes mentioned. This level of detail is useful in understanding user opinions about various features of a product. In this study, ABSA was employed to analyze user reviews of laptops from Twitter, aiming to extract sentiments associated with specific aspects such as “battery,” “screen,” and “price” to gain insights into user satisfaction across seven major brands.

The implementation of ABSA was facilitated by the PyABSA library, a Python-based framework that provides pre-trained models and utilities for aspect extraction and sentiment prediction. The analysis was conducted using Google Colab, a cloud-based platform offering free computational resources, supporting Python code execution, and providing the necessary tools for machine learning tasks.

Applying the ABSA methodology allowed the identification of various aspects mentioned in reviews and the classification of sentiments associated with each as positive, negative, or neutral. This granular sentiment analysis provided a detailed understanding of user opinions about different laptop features, essential for the subsequent steps of the study.

Aspect extraction, a fundamental step in ABSA, involves identifying specific attributes or features mentioned in reviews and determining the associated sentiment. This detailed analysis offers insights into user opinions about features such as “battery,” “RAM,” and “price.” For instance, in the review “Super great laptop even though it had 8 GB of RAM, it is also very thin and lightweight, and the battery lasts a long time,” the identified aspects were “RAM,” “weight,” and “battery,” with corresponding sentiments being “Neutral,” “Positive,” and “Positive,” respectively.

The aspect extraction process involved using the PyABSA library to extract aspects and associated sentiments from each review, analyzing the frequency of each aspect to determine the most commonly mentioned features, and creating a target aspect list based on frequency analysis. The final list of target aspects included [‘ram’, ‘display’, ‘keyboard’, ‘price’, ‘battery’, ‘storage’, ‘gpu’, ‘cpu’, ‘charger’, ‘warranty’, ‘design’, ‘webcam’, ‘port’, ‘size’, ‘fan’, ‘weight’].

Challenges such as synonyms and variants were addressed by normalizing terms to ensure consistency. The final aspect list served as a basis for analyzing sentiments associated with each aspect in the reviews, providing a comprehensive analysis of user opinions regarding critical laptop features and forming the foundation for understanding user satisfaction and identifying areas for improvement.

The result involved summarizing the sentiments associated with each extracted aspect, focusing on the most frequently mentioned ones to gain a comprehensive understanding of user opinions about different laptop features. Sentiments were categorized as positive or negative, and radar and butterfly charts were plotted to visualize the overall sentiment distribution for each aspect, allowing for a quick comparison of sentiment intensity across different aspects.

**Fig 4:**
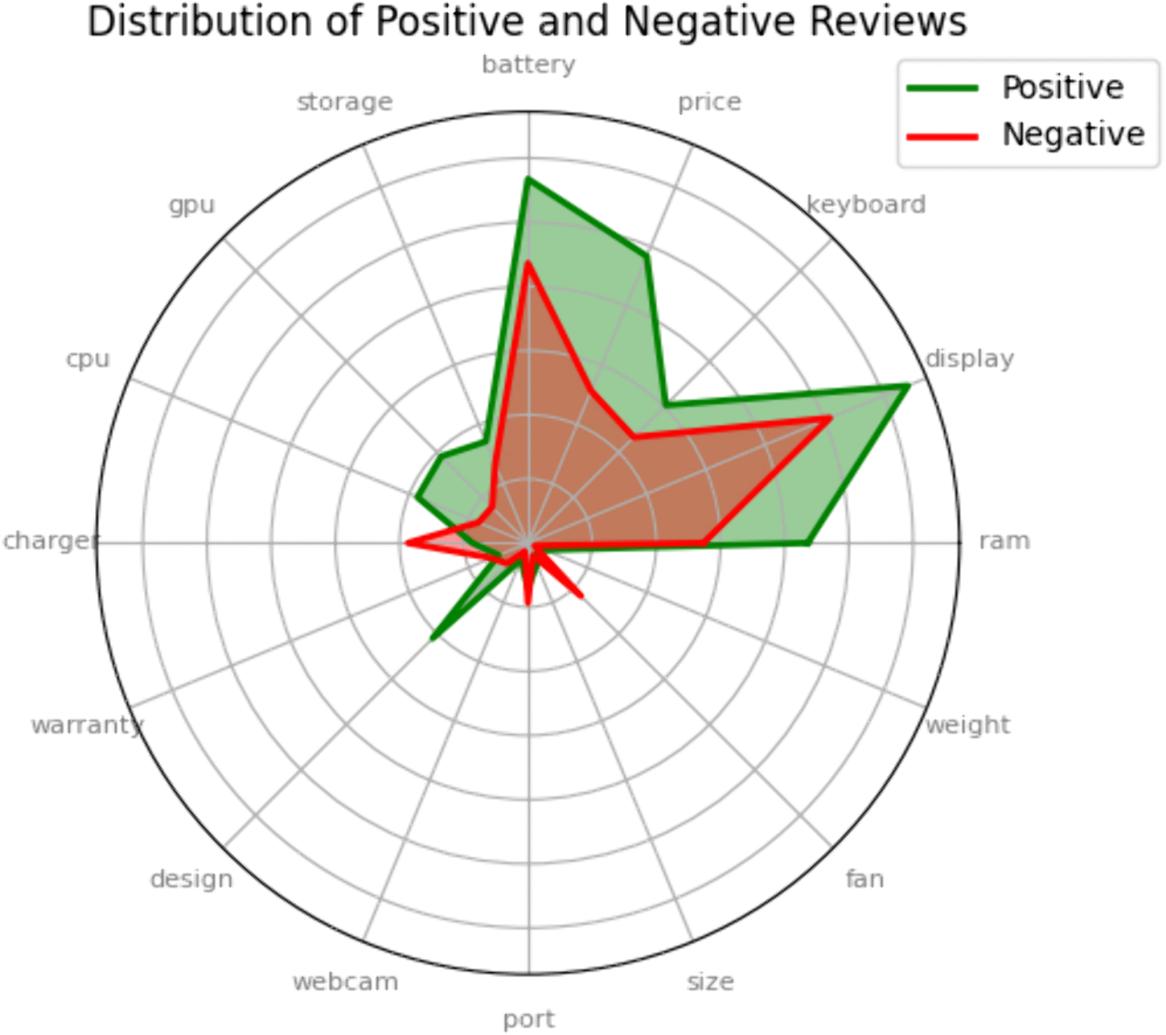
Distribution of Positive and Negative reviews.

Fig 4 offers a comprehensive visualization of user sentiments across various laptop features. High positive sentiment is noted in aspects such as design, with 82.2% positive feedback, weight at 72.5%, GPU with 70.7%, and CPU at 69.0%. Conversely, the fan shows an 85.1% negativity rate, and the charger faces 68.33% negative reviews, indicating common issues with cooling efficiency, battery charging.

Balanced sentiments are observed in features like RAM, with 61.4% positive feedback, display at 55.7%, keyboard at 56.6%, and storage at 56.2%. These features generally meet user expectations but suggest areas where small improvements could enhance satisfaction. The display and battery, which attract the most reviews, are crucial features for users, underscoring their significant role in the overall user experience. Additionally, the price, with a 65.3% positivity rate, reflects user sensitivity to cost versus value, emphasizing the importance of competitive pricing strategies.

After conducting a comprehensive analysis of the overall dataset, we now shift our focus to a more detailed examination of individual laptop brands. By analyzing user sentiments for each brand, we can uncover unique insights and brand-specific trends that might not be visible in the aggregate data. This targeted analysis will deepen our understanding of consumer preferences and pain points for each brand, providing valuable guidance for targeted improvements and strategic marketing initiatives.

**Fig 5:**
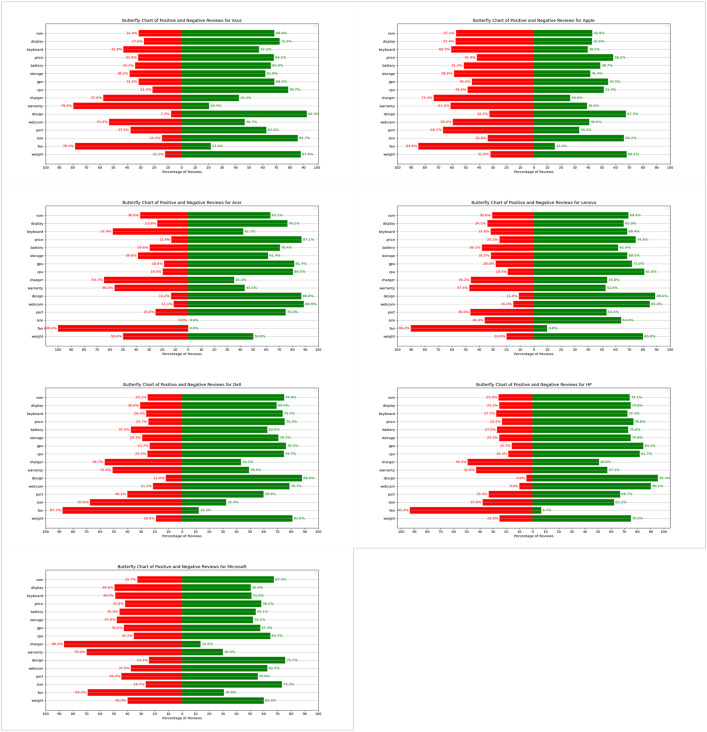
Brand-specific sentiment patterns across seven notebook manufacturers revealing unique strengths and weaknesses per brand.

1. **Asus:** Asus laptops have garnered significant user sentiment, highlighting both strengths and areas for improvement. The design stands out with an impressive 92.3% positive feedback, underscoring its aesthetic appeal and ergonomic excellence (Fig 5). Users also highly appreciate the lightweight and portable designs (87.9% positive sentiment). In terms of performance, Asus laptops receive strong approval for their CPU (78.7% positive) and GPU (68.5% positive), showcasing robust processing and graphics capabilities. However, there are notable concerns regarding the fan system (78.4% negative sentiment), potentially affecting cooling efficiency. The charger (57.6% negative) also drew criticism, primarily due to battery charging reliability issues. Warranty services received considerable negative feedback (79.6% negative), indicating room for improvement in customer support.
2. Acer: User sentiments towards Acer laptops reveal a mixed bag of strengths and challenges. Battery life stands out with a prominent 70.4% positive sentiment, reflecting high user satisfaction (Fig 5). The display quality is another highlight, praised for its visual excellence (76.2% positive). Acer excels in GPU (81.4% positive) and CPU (80.5% positive) performance, demonstrating powerful processing capabilities. However, concerns about the fan system are prevalent (almost 100% negative sentiment), indicating significant cooling issues. Dissatisfaction with the charger (64.7% negative) and warranty (56.5% negative) further highlights reliability issues. The keyboard also receives notable criticism (57.8% negative), affecting usability and comfort.
3. Apple: Apple laptops are celebrated for their distinct features, though not without critical feedback. Design is a standout feature with a notable 67.5% positivity rate, emphasizing aesthetics and ergonomic appeal (Fig 5). Weight is also positively received (68.1% positive), contributing to the laptops’ portability. However, concerns about the fan (84.6% negative sentiment), charger (73.4% negative), keyboard (60.5% negative), and warranty (61.0% negative) indicate significant areas for improvement.
4. Dell: Dell laptops are well-regarded for their overall performance and design appeal (Fig 5). Design receives an impressive 88.0% positive feedback, reflecting its aesthetic and ergonomic strengths. Weight is another positive aspect (81.0% positive), contributing to its portability. Performance-wise, Dell laptops excel in GPU (76.3% positive) and CPU (74.7% positive) capabilities. However, concerns about the fan system (87.7% negative sentiment), and charger (56.7% negative) underscore areas needing improvement.
5. HP: HP laptops earn high praise for their design (95.4% positive sentiment), emphasizing aesthetic and ergonomic qualities (Fig 5). Weight is also positively received (75.0% positive), contributing to their portability. Performance aspects such as CPU (81.7% positive) and GPU (84.3% positive) capabilities are well-regarded. However, critical feedback regarding the fan (93.3% negative sentiment) indicates areas needing attention.
6. Lenovo: User sentiments towards Lenovo laptops highlight strong design appeal (89.0% positive sentiment), emphasizing aesthetics and ergonomic considerations (Fig 5). Weight is also positively received (80.0% positive), contributing to their portability. Performance-wise, Lenovo excels in CPU (81.0% positive) and GPU (72.0% positive) capabilities. However, concerns about the fan system (90.2% negative sentiment) indicate potential areas for improvement.
7. Microsoft: Microsoft laptops receive commendation for their design (75.7% positive sentiment), highlighting aesthetic and ergonomic features (Fig 5). Performance aspects such as CPU (64.7% positive) and GPU (57.4% positive) capabilities show strong approval. However, there are significant concerns about the charger (86.5% negative sentiment), and warranty (70.0% negative), indicating areas needing improvement.

### 4.4. Determining the importance of sentiments in notebook selection

After identifying users’ sentimental responses to various features and aspects of notebooks, it is crucial to understand how these sentiments influence the selection and purchase of laptops. To gain an accurate and practical understanding of this phenomenon, we consulted industry experts with direct experience in notebook sales. These experts don’t replace consumer preferences but rather interpret them through their accumulated experience with actual purchase patterns. They understand the difference between features consumers complain about online versus features that make them walk away from a purchase. This practical knowledge helps weight the importance of sentiments in real purchasing contexts. Using the snowball sampling method, we selected 10 notebook sales experts from diverse backgrounds. Table 3 presents the demographic and professional profiles of these experts.

**Table 3:** Profile of experts.

ID	Gender	Country	Experience (years)	Position	Store Type
E1	Male	Germany	16	Sales Manager	Laptop store
E2	Female	Canada	10	Salesperson	Laptop store
E3	Male	South Korea	12	Marketing Manager	Laptop store
E4	Female	Spain	8	Salesperson	Laptop store
E5	Male	Japan	11	Senior Sales Manager	Digital goods store
E6	Male	France	14	Marketing Manager	Digital goods store
E7	Female	Brazil	9	Salesperson	Digital goods store
E8	Female	China	10	Sales Manager	Digital goods store
E9	Female	England	11	Marketing Manager	Digital goods store
E10	Male	Australia	13	Sales Manager	Laptop store

As shown in Table 3, our expert panel comprises professionals in key decision-making positions including Sales Managers, Marketing Managers, and Senior Sales personnel who directly influence and observe consumer purchasing patterns. These positions provide them with unique insights into the gap between expressed consumer sentiments and actual purchase behaviors. The diversity in their roles, from frontline salespersons who interact directly with customers to marketing managers who analyze purchase trends, ensures comprehensive coverage of the consumer decision-making process.

The objective of this stage is to determine, through industry experts, the extent to which positive sentiments towards specific notebook features motivate purchase decisions, and conversely, how negative sentiments may deter customers from buying a particular product. To achieve this, we designed an online questionnaire for our panel of experts. The survey asked them to rate, using linguistic terms commonly employed in fuzzy logic for data collection (very low, low, medium, high, very high), the impact of both positive and negative sentiments across 16 notebook features. Specifically, they were asked to evaluate how positive sentiments create motivation for purchase and how negative sentiments create barriers to purchase. Table 4 presents the consolidated results of this questionnaire.

**Table 4:** The results of expert opinions.

Positive Sentiment	Laptop Feature	E1	E2	E3	E4	E5	E6	E7	E8	E9	E10
**RAM**	High	High	Very High	High	Very High	High	High	Very High	High	High
**Display**	High	Very High	Very High	Very High	High	Very High	Very High	High	Very High	High
**Keyboard**	Medium	Medium	Medium	Medium	Medium	Medium	Medium	Medium	Medium	Medium
**Price**	Very High	Very High	Very High	Very High	Very High	Very High	Very High	Very High	Very High	Very High
**Battery**	High	High	High	Medium	High	High	High	High	High	High
**Storage**	High	High	High	High	High	High	High	High	High	High
**GPU**	Medium	Medium	High	Medium	High	Medium	Medium	High	Medium	Medium
**CPU**	High	High	Very High	High	Very High	High	High	Very High	High	High
**Charger**	Low	Low	Low	Low	Low	Low	Low	Low	Low	Low
**Warranty**	Medium	High	Medium	Medium	High	High	Medium	Medium	High	High
**Design**	Medium	High	Very High	High	High	Very High	High	High	Very High	High
**Webcam**	Low	Medium	Medium	Low	Medium	Medium	Medium	Medium	Medium	Low
**Port**	Medium	Medium	Medium	Medium	Medium	Medium	Medium	Medium	Medium	Medium
**Size**	Medium	High	High	High	Medium	High	High	Medium	High	Medium
**Fan**	Low	Low	Low	Low	Low	Low	Low	Low	Low	Low
**Weight**	Medium	High	High	High	Medium	High	High	Medium	High	Medium
Negative Sentiment	**RAM**	High	High	Very High	High	Very High	High	High	Very High	High	High
	**Display**	High	Very High	Very High	High	High	Very High	High	High	Very High	High
	**Keyboard**	Medium	Medium	Medium	Medium	Medium	Medium	Medium	Medium	Medium	Medium
	**Price**	Very High	Very High	Very High	Very High	Very High	Very High	Very High	Very High	Very High	Very High
	**Battery**	High	High	High	Medium	High	High	High	High	High	High
	**Storage**	High	High	High	High	High	High	High	High	High	High
	**GPU**	Medium	Medium	High	Medium	Medium	Medium	Medium	High	Medium	Medium
	**CPU**	High	High	Very High	High	Very High	High	High	Very High	High	High
	**Charger**	Low	Low	Low	Low	Low	Low	Low	Low	Low	Low
	**Warranty**	Medium	High	Medium	Medium	High	High	Medium	Medium	High	High
	**Design**	Medium	Medium	High	Medium	Medium	High	Medium	Medium	High	Medium
	**Webcam**	Low	Low	Low	Low	Low	Low	Low	Low	Low	Low
	**Port**	Medium	Medium	Medium	Medium	Medium	Medium	Medium	Medium	Medium	Medium
	**Size**	Medium	Medium	Medium	Medium	Medium	Medium	Medium	Medium	Medium	Medium
	**Fan**	Low	Low	Low	Low	Low	Low	Low	Low	Low	Low
	**Weight**	Medium	Medium	Medium	Medium	High	Medium	Medium	Medium	Medium	Medium

To defuzzify the expert opinions provided in Table 4, the center of gravity (COG) method was employed. This method converts linguistic terms into precise numerical values using fuzzy logic principles, facilitating a quantitative analysis of qualitative data. The fuzzy numbers assigned to each linguistic term are as Table 5:

**Table 5:** Linguistic scales.

Linguistic Scale	Fuzzy Number	Defuzzification values
Very low (VL)	(0, 0, 0.25)	0.0833
Low (L)	(0, 0.25, 0.5)	0.25
Medium (M)	(0.25, 0.5, 0.75)	0.5
High (H)	(0.5, 0.75, 1)	0.75
Very high (VH)	(0.75, 1, 1)	0.9167

By applying these values, the linguistic ratings from Table 4 were transformed into numerical values. Subsequently, the average score for each notebook feature was calculated for both positive and negative sentiments. The resulting averages for each feature are presented in Table 6, which indicates the degree to which positive and negative sentiments influence the purchase decision for each feature:

**Table 6:** Average Defuzzification values.

notebook Feature	Positive Sentiment Average	Negative Sentiment Average
RAM	0.8333	0.8333
Display	0.8833	0.85
Keyboard	0.5	0.5
Price	0.9167	0.9167
Battery	0.75	0.75
Storage	0.75	0.75
GPU	0.6083	0.6
CPU	0.8333	0.8333
Charger	0.25	0.25
Warranty	0.6667	0.6667
Design	0.7833	0.6
Webcam	0.3583	0.25
Port	0.5	0.5
Size	0.6333	0.5
Fan	0.25	0.25
Weight	0.6333	0.55

The results of the defuzzification process, derived from the expert opinions of notebook sales professionals, provide valuable insights into which notebook features significantly influence purchase decisions through positive and negative sentiments. Features such as price, display, and RAM exhibit high average scores for both positive and negative sentiments (0.9167, 0.8833, and 0.8333 respectively), indicating that these features are crucial in motivating customers to buy notebooks and, conversely, can act as significant barriers if perceived negatively. The high scores for these features suggest that customers place considerable importance on the performance and cost-effectiveness of the notebook, and any dissatisfaction in these areas can greatly deter potential buyers. On the other hand, features such as the charger, and fan have notably lower average scores for both positive and negative sentiments (both 0.25, 0.25), implying that these aspects are less critical in influencing purchase decisions. This suggests that while these features are part of the overall product package, they do not play a major role in the customer’s motivation to purchase a notebook. Additionally, the relatively moderate scores for features like the keyboard, webcam, and port (0.5, 0.3583, and 0.5 respectively) indicate that while these factors are important, they are not as pivotal as the core performance and design features.

### 4.5. TOPSIS-based prioritization of notebook features

The Technique for Order of Preference by Similarity to Ideal Solution (TOPSIS) represents the synthesis stage of our hybrid framework, where consumer sentiments and expert knowledge converge to generate strategic priorities. This integration addresses a fundamental challenge in market analysis: the disconnect between sentiment intensity and purchase influence.

The TOPSIS methodology serves three critical functions in our framework. First, it transforms multi-dimensional sentiment data into a unified decision matrix by incorporating both positive and negative sentiment percentages for each feature. Second, it weights these sentiments according to expert-validated importance scores, acknowledging that consumer emotions do not uniformly translate into purchase behaviors. Third, it calculates the relative closeness of each feature to ideal and negative-ideal solutions, producing a comprehensive ranking that balances consumer preferences with market realities.

This methodological integration reveals that features generating strong emotional responses may not necessarily drive purchase decisions, while features with moderate sentiments can be critical determinants of consumer choice. The TOPSIS analysis thus bridges the gap between what consumers express (captured through ABSA) and what actually influences their purchasing behavior (validated through expert knowledge), providing manufacturers with actionable priorities that reflect both market sentiment and purchase psychology.

Preparation and Implementation of TOPSIS Analysis:

1. **Decision Matrix Construction:** A matrix was created with notebook features (e.g., RAM, Display, CPU) as rows and four columns:
  a) Positive sentiment scores from social media analysis
  b) Negative sentiment scores from social media analysis
  c) Positive sentiment weights from expert evaluations
  d) Negative sentiment weights from expert evaluations

This structure allows for a holistic view of each feature, considering both consumer opinions and expert assessments.

2. **Normalization:** To ensure fair comparison across different scales, the decision matrix was normalized. This step is crucial as it converts all values to a common scale, typically between 0 and 1.
3. **Weighted Normalization:** The normalized matrix was then weighted using the expert-derived importance scores. This step emphasizes the relative importance of each criterion as determined by industry professionals.
4. **Ideal Solutions Identification:** Two hypothetical solutions were defined:
  - Positive Ideal Solution (PIS): Representing the best possible scenario with maximized positive sentiments and minimized negative sentiments.
  - Negative Ideal Solution (NIS): Representing the worst possible scenario with minimized positive sentiments and maximized negative sentiments.
5. **Distance Calculation:** Euclidean distances were calculated from each notebook feature to both the PIS and NIS. This step quantifies how close each feature is to the ideal and worst-case scenarios.
6. **Relative Closeness Calculation:** The relative closeness of each feature to the ideal solution was computed. This value, ranging from 0 to 1, indicates how well a feature performs overall, considering both its proximity to the ideal solution and its distance from the worst solution.
7. **Ranking:** Finally, notebook features were ranked based on their relative closeness values. Features with higher values are considered more desirable, as they are closer to the ideal solution and farther from the negative ideal solution.

The results obtained from TOPSIS calculations are detailed in Table 7, which delineates a distinct hierarchy of consumer preferences. Key findings highlight that price, design, display, CPU, and RAM are paramount factors influencing consumer choice in notebooks. These top-ranked attributes underscore consumer preferences for a blend of affordability, aesthetic appeal, visual quality, and core performance when selecting their purchases.

**Table 7:** TOPSIS-based prioritization ranking of 16 notebook features.

Rank	Notebook Feature	TOPSIS Score
1	Price	0.6266
2	Design	0.5894
3	Display	0.589
4	CPU	0.5731
5	RAM	0.561
6	Storage	0.4998
7	Battery	0.4996
8	Warranty	0.4598
9	Weight	0.4292
10	GPU	0.4118
11	Size	0.3906
12	Port	0.3271
13	Keyboard	0.3122
14	Webcam	0.2208
15	Fan	0.1723
16	Charger	0.1359

To capitalize on these insights, companies should tailor their marketing strategies to accentuate competitive pricing, stylish designs, high-quality displays, and robust processing capabilities. By emphasizing these strengths, marketing campaigns can effectively communicate the value propositions of their products, showcasing how they excel in critical areas that resonate most with consumers. Conversely, features such as webcam, fan, and charger ranked lowest in priority, indicating their minimal impact on consumer decision-making. While essential components, their lower ranking suggests they do not significantly differentiate products in the current market. Nonetheless, this highlights an opportunity for innovation. Companies can invest in research and development to reimagine these features, potentially enhancing overall product appeal by adding unexpected value to the user experience.

Aligning strategies with these findings enables notebook manufacturers to optimize resource allocation. They can focus marketing efforts on highlighting strengths in high-priority areas, potentially boosting market appeal and sales. Simultaneously, directing R&D towards improving lower-ranked features can lead to innovative solutions that differentiate products in a competitive market landscape.

## 5. Discussion

The integration of ABSA and TOPSIS methodologies in this study provides a more nuanced understanding of the notebook market than either approach could achieve independently. While ABSA captures the raw emotional responses of consumers toward specific features, it cannot distinguish between sentiments that merely express preferences and those that drive actual purchase decisions. TOPSIS addresses this limitation by incorporating expert knowledge to weight the relative importance of different sentiments. This methodological synthesis reveals critical market insights: features that generate strong emotional responses do not necessarily influence purchase decisions proportionally, and conversely, features with moderate sentiment scores may be decisive factors in consumer choice. This integrated approach enables manufacturers to avoid misallocating resources based solely on sentiment intensity and instead focus on features that genuinely drive market behavior.

The complementary nature of these methodologies becomes evident when examining specific findings. ABSA identified genuine consumer pain points such as cooling systems and battery chargers with predominantly negative sentiments, while TOPSIS contextualized these findings by revealing that despite strong negative responses, these features have minimal impact on purchase decisions compared to core attributes like price and display quality. This dual-perspective analysis prevents manufacturers from overinvesting in addressing issues that, while generating user frustration, do not significantly influence purchasing behavior.

The TOPSIS prioritization results presented in Table 8 reveal important insights about brand-specific strengths and market positioning. Rather than suggesting all brands should pursue identical strategies, these results highlight each brand’s unique competitive advantages and natural differentiation opportunities.

**Table 8:** The results of TOPSIS prioritization by brands.

Rank	Acer	Apple	Asus	Dell	HP	Lenovo	Microsoft
1	RAM	Price	Price	Price	Price	Display	Price
2	Display	Display	Display	Display	Display	Price	Display
3	Price	CPU	RAM	CPU	RAM	RAM	CPU
4	CPU	RAM	CPU	RAM	CPU	CPU	RAM
5	Storage	Storage	Storage	Battery	Battery	Battery	Storage
6	Battery	Battery	Battery	Storage	Storage	Storage	Battery
7	Warranty	Warranty	Warranty	Warranty	Design	Design	Warranty
8	Design	Design	Design	Design	Warranty	Warranty	Design
9	Size	GPU	Weight	Weight	Weight	Weight	GPU
10	Weight	Weight	GPU	Size	GPU	GPU	Weight
11	GPU	Size	Size	GPU	Size	Size	Size
12	Keyboard	Port	Keyboard	Port	Port	Port	Keyboard
13	Port	Keyboard	Port	Keyboard	Keyboard	Keyboard	Port
14	Fan	Fan	Fan	Fan	Webcam	Webcam	Charger
15	Webcam	Charger	Webcam	Webcam	Fan	Fan	Webcam
16	Charger	Webcam	Charger	Charger	Charger	Charger	Fan

For premium positioning, Apple and Microsoft show distinct patterns where design and display quality rank highly, aligning with their focus on creative professionals and business users. Apple’s lower emphasis on price (ranking 2nd) reflects its established premium brand equity, while Microsoft’s similar pattern supports its Surface line’s positioning as productivity-focused devices.

In the value segment, brands like Acer and Asus show price and RAM as top priorities, consistent with their strategy of offering performance-oriented machines at competitive prices. Acer’s top ranking of RAM reflects its gaming laptop focus, while Asus balances price with display quality to serve both budget-conscious and enthusiast markets.

For business-oriented brands, Lenovo’s unique prioritization of display quality over price aligns with its ThinkPad heritage of serving professional users who value screen quality for extended work sessions. Dell and HP show similar patterns with price leading, reflecting their broad market approach spanning from entry-level to premium segments.

These brand-specific patterns suggest that rather than converging on a single “ideal” notebook profile, manufacturers should leverage their existing strengths. Lenovo should continue emphasizing its superior displays, Apple should maintain its design excellence, and value brands should focus on delivering performance at attractive price points. The data supports differentiation rather than homogenization.

In conclusion, the TOPSIS prioritization results offer a data-driven framework for notebook manufacturers to align their marketing and R&D strategies with consumer preferences. By emphasizing their strengths in high-priority areas through targeted marketing campaigns and addressing weaknesses through focused innovation, companies can potentially enhance their market position. However, this approach should be complemented by a holistic understanding of market dynamics, consumer behavior, and brand-specific factors to develop truly effective and sustainable strategies in the highly competitive notebook industry.

### 5.1. Managerial Insights and Implementation

The findings of this research offer valuable insights for notebook manufacturers and marketers, providing a data-driven approach to enhance their competitive positioning in the market. The TOPSIS prioritization results, coupled with sentiment analysis from social media and expert evaluations, present a comprehensive framework for strategic decision-making within each brand’s unique market context.

The analysis reveals that while certain features consistently rank highly across brands, such as price, display quality, and core performance components, the specific prioritization patterns align with and validate each brand’s existing market positioning. This differentiation is not a weakness to be corrected but rather a strength that reflects the diverse needs of the notebook market. For instance, Apple’s emphasis on design excellence serves creative professionals, Lenovo’s display quality focus aligns with business users’ extended work sessions, while Acer’s RAM prioritization supports gaming enthusiasts’ performance needs.

Given these brand-specific patterns, manufacturers should interpret our findings as optimization guidance within their chosen market segments rather than universal prescriptions. The following insights should be adapted to each brand’s strategic context:

Regarding pricing strategies, while price ranks consistently high, its implementation varies by segment. Premium brands like Apple can maintain higher price points justified by design and ecosystem integration, while value-focused brands like Acer and Asus should continue optimizing cost structures to offer competitive pricing without compromising their performance advantages.

For display technology, the high consumer priority suggests all brands should invest in this area, but through different approaches. Business-oriented brands might focus on color accuracy and eye-comfort technologies, gaming brands on refresh rates and response times, while creative-focused brands on color gamut and resolution.

Core performance features (RAM, CPU) remain fundamental, but their emphasis should align with use cases. Gaming and performance brands should pursue cutting-edge specifications, while ultrabook manufacturers might balance performance with efficiency and thermal management.

The consistently low ranking of features like webcams, fans, and chargers presents differentiation opportunities precisely because they are overlooked. A brand could distinguish itself by transforming these “hygiene factors” into competitive advantages, for instance, superior cooling enabling sustained performance, or professional-grade webcams for the remote work era.

To effectively implement these strategies within their market positions, manufacturers should:

I. Regularly conduct sentiment analysis on social media platforms, filtering insights by their target demographic rather than the general market.
II. Establish cross-functional teams that understand both the brand’s strategic positioning and evolving consumer preferences within their segment.
III. Develop product roadmaps that strengthen existing brand advantages while selectively addressing weaknesses that matter to their specific consumer base.
IV. Create marketing campaigns that resonate with their target segment’s priorities rather than attempting to appeal to all consumers.
V. Monitor both direct competitors within their segment and potential disruptors from adjacent segments.

By leveraging these insights through the lens of their established market positions, notebook manufacturers can strengthen their differentiation, better serve their target consumers, and maintain sustainable competitive advantages in distinct market segments.

## 6. Conclusion

This research successfully developed and validated a novel hybrid framework for analyzing consumer preferences in the notebook market by integrating three key components: aspect-based sentiment analysis of social media data, expert evaluations through fuzzy logic, and feature prioritization using TOPSIS methodology. The framework effectively addressed the primary research objectives by establishing a systematic approach for real-time analysis of consumer sentiments across multiple notebook features, quantifying expert knowledge through fuzzy logic implementation, and developing a reproducible method for feature prioritization across different brands.

The study’s empirical validation, analyzing 329,091 tweets across seven major brands (Asus, Acer, Apple, Dell, HP, Lenovo, and Microsoft), demonstrated the framework’s effectiveness in identifying and ranking consumer preferences. The ABSA component successfully extracted sentiments toward 16 key notebook attributes, revealing that design, weight, GPU, and CPU received predominantly positive feedback, while cooling systems and battery chargers emerged as primary pain points across brands. The integration of expert evaluations through fuzzy logic provided crucial context about which sentiments translate into actual purchasing behavior, bridging the gap between stated and revealed consumer preferences.

The TOPSIS-based prioritization revealed that price, display quality, CPU performance, RAM capacity, and design constitute the most influential factors in notebook purchasing decisions. Importantly, the brand-specific analysis demonstrated distinct prioritization patterns that align with each manufacturer’s market positioning: Lenovo’s emphasis on display quality reflects its business-oriented focus, Apple’s design prominence aligns with its creative professional target market, and value-focused brands like Acer and Asus show price and RAM as top priorities consistent with their performance-oriented strategies.

The methodology provides manufacturers with a quantifiable, actionable framework for optimizing product development and marketing strategies. By synthesizing consumer voice captured through social media with expert knowledge of purchase behavior, the framework enables companies to prioritize genuinely purchase-influencing features while addressing critical pain points. The open availability of the dataset on Hugging Face and analytical code on GitHub ensures reproducibility and facilitates future research extensions.

This hybrid approach contributes to both academic literature and industry practice by demonstrating how machine learning-based sentiment analysis can be systematically integrated with structured expert knowledge and multi-criteria decision-making methods. The framework offers a comprehensive blueprint for understanding consumer preferences in dynamic technology markets and can serve as a foundation for similar analyses in other consumer electronics sectors.

## 7. Limitations and future research

While this research provides valuable insights into consumer preferences and brand positioning in the notebook market, several limitations warrant acknowledgment. The study primarily relied on social media data from X (formerly Twitter), which may not fully represent the entire consumer base. Users of this platform may have specific demographic characteristics or usage patterns that could skew the results. Twitter users tend to be younger, more technologically engaged, and potentially more vocal about their opinions compared to the broader consumer population [86,87]. This demographic concentration could introduce selection bias, potentially overrepresenting certain viewpoints while underrepresenting others, such as older consumers or those less active on social media platforms. Furthermore, the non-deterministic nature of the X API presents inherent limitations, as different data collection iterations may yield varying tweet samples even with identical search parameters. However, our large sample size (329,091 tweets) and extended collection period (18 months) help mitigate the impact of such variations on overall findings.

The research focused on a specific time frame (January 2023 to June 2024), which may not capture long-term trends or seasonal variations in consumer sentiment. The notebook market is characterized by rapid technological advancement, with new product releases, operating system updates, and component innovations occurring frequently. Consumer preferences may shift substantially in response to disruptive innovations such as the introduction of new processor architectures, display technologies, or form factors. Additionally, seasonal patterns related to academic calendars, holiday shopping periods, and corporate purchasing cycles may influence sentiment expressions in ways not fully captured by our study period.

The TOPSIS method, while robust and widely validated in multi-criteria decision-making contexts, assumes independence among criteria. However, notebook features such as CPU performance, RAM capacity, and overall system responsiveness are inherently interrelated [88]. Similarly, design aesthetics and weight considerations often involve trade-offs, as do battery life and processing power. This assumption of independence represents a theoretical simplification that may not fully capture the complex interdependencies and compensatory relationships among notebook attributes. The expert panel, while diverse in terms of geographic representation and professional roles, was limited to 10 professionals. Although this sample size aligns with established practices in fuzzy multi-criteria decision-making studies, it may constrain the breadth of industry insights, particularly regarding specialized market segments such as gaming, professional creative workstations, or enterprise deployments.

Future research could address these limitations through several approaches. A more comprehensive study could incorporate data from multiple social media platforms, specialized technology forums, and e-commerce review sites to provide a more representative sample of consumer opinions. This multi-source approach would capture diverse demographic segments and usage contexts while enabling comparative analysis across different platforms and communities. Longitudinal studies tracking sentiment evolution over extended periods (3–5 years) would enable identification of long-term trends and technological adoption patterns, while real-time sentiment monitoring systems could provide dynamic insights for rapid response to emerging issues or opportunities.

Methodologically, exploring alternative multi-criteria decision-making methods that explicitly model feature interdependencies, such as Analytic Network Process (ANP) or Decision-Making Trial and Evaluation Laboratory (DEMATEL), would address the independence assumption limitation. Integration of machine learning techniques for dynamic feature weighting based on evolving market conditions could enhance adaptability, while hybrid approaches combining conjoint analysis with sentiment analysis could provide deeper insights into feature trade-offs and compensatory decision-making patterns. Enlarging and diversifying the expert panel to include product designers, user experience researchers, and supply chain professionals would enrich the knowledge base, while incorporating direct consumer interviews or focus groups would strengthen the validation of social media findings.

Cross-cultural studies examining how consumer preferences vary across different geographic markets and economic development levels would reveal global patterns, while research segmenting consumers by use case (students, professionals, gamers, content creators) would enable more targeted strategies. Investigating consumer sentiments toward emerging notebook features such as AI-enhanced capabilities, sustainability considerations, modular designs, and repairability would prepare manufacturers for future market shifts. Finally, validation studies comparing sentiment-based predictions with actual sales data and market share changes would establish the predictive validity of this framework, while benchmarking against traditional market research methods would clarify the relative advantages and limitations of social media-based approaches.

Through systematic investigation of these directions, researchers can enhance our understanding of consumer preferences in rapidly evolving technology markets while addressing the inherent limitations of current methodologies and developing more robust frameworks for strategic decision-making.
